# Diferencias por sexo y determinantes en la realización de la prueba de cribado de cáncer colorrectal en España (2009-2023)

**DOI:** 10.23938/ASSN.1143

**Published:** 2025-10-29

**Authors:** Maria Gisbert Canet, Nayara Tamayo Fonseca, Javier Casillas Clot, Pamela Pereyra Zamora, Andreu Nolasco

**Affiliations:** Universidad de Alicante Departamento de Enfermería Comunitaria, Medicina Preventiva y Salud Pública e Historia de la Ciencia Alicante España

**Keywords:** Cribado sistemático, Cáncer Colorrectal, Determinantes Sociales de la Salud, Encuesta de Salud, Mass Screenings, Colorectal Cancer, Social Determinants of Health, Health Survey

## Abstract

**Fundamento:**

Describir las diferencias por sexo y por determinantes sociales de la salud en la evolución de la adhesión al cribado de cáncer colorrectal en España (2009-2023).

**Metodología:**

Estudio transversal realizado en España con datos de las encuestas de salud europeas (2009-2014-2020) y española (2023). Se analizó la frecuencia de realización de la prueba de sangre oculta en heces en personas de 50-69 años, por comunidades autónomas y por variables relativas a determinantes sociales de la salud, mediante Chi-cuadrado. Las tendencias temporales se analizaron mediante regresión *joinpoint*.

**Resultados:**

Entre 2009 y 2023 se observó un aumento generalizado de la adhesión al programa de cribado, con diferencias entre comunidades (p<0,005). La adherencia de algunas de las comunidades con mayor antigüedad en el cribado descendió en 2023. En 2014 se identificó un punto de cambio en la tendencia temporal: el porcentaje anual de cambio descendió del 33,6% al 10,0% (p<0,005). La existencia de programa de cribado, sexo masculino, mayor edad, niveles altos de estudios e ingresos, convivencia, estilo de vida saludable, peor estado de salud o problemas en el entorno urbano se asociaron con mayor realización de la prueba.

**Conclusiones:**

La pandemia de COVID-19 podría haber afectado a los programas de cribado. De mantenerse el porcentaje de cambio anual estimado, el objetivo de participación establecido por la Estrategia en Cáncer española podría alcanzarse al final de esta década. Es esencial profundizar en los análisis con perspectiva de género interseccional para abordar la diferente participación en mujeres y hombres.

## Abstract (en)

**Background:**

This study aimed to examine sex differences and the influence of social determinants of health on the uptake of colorectal screening in Spain from 2009 to 2023.

**Methods:**

A cross-sectional analysis was conducted using data from the European Health Interview Survey in Spain (2009, 2014, 2020) and the Spanish Health Survey 2023. The frequency of faecal occult blood test use among individuals aged 50 to 69 was analysed by autonomous community and by variables related to social determinants of health using Chi-square tests. Temporal trends were assessed through join point regression.

**Results:**

Overall, faecal occult blood test uptake increased during the study period, with significant variation across autonomous communities (p<0.005). In 2023, some communities with longer-standing screening programs showed a decline in participation. A join point was identified in 2014, with the annual percentage change decreasing from 33.6% to 10.0% (p<0.005). Higher test utilization was associated with the presence of a screening program, male sex, older age, higher education and income levels, cohabitation, healthy lifestyle behaviours, poorer health status, and urban residence.

**Conclusions:**

The COVID-19 pandemic may have substantially affected screening participation. If the observed annual percentage of change is maintained, participation targets may be reached by the end of this decade. Further analyses incorporating an intersectional gender perspective are essential to understand and address the observed differences in participation between women and men.

## INTRODUCCIÓN

En España, el cáncer es la primera causa de muerte en hombres y la segunda en mujeres^1^; el más frecuente en ambos sexos es el colorrectal (CCR)^2^. Los casos de cáncer han aumentado en las últimas décadas debido al envejecimiento poblacional, la mayor exposición a factores de riesgo y una mayor detección precoz mediante cribados poblacionales^1^^,^^3^. Paralelamente, la mortalidad ha disminuido gracias a la prevención y los avances terapéuticos^1^. No obstante, en España, la reducción de los factores de riesgo modificables sigue suponiendo un reto^1^. Entre los factores relacionados con el desarrollo de CCR se encuentran la edad, el origen afroamericano, la obesidad, el sedentarismo, la dieta rica en carne procesada, el tabaco y el alcohol, además de factores hereditarios. Son protectores la actividad física y la dieta rica en verdura, fruta o cereales integrales^4^. Participar en programas organizados de cribado también reduce el riesgo^5^.

Actualmente, el programa de cribado poblacional de CCR está implantado en todo el territorio español, si bien la fecha de inicio en cada comunidad varía (Anexo I)^6^. La participación difiere considerablemente entre comunidades autónomas (entre el 19% y el 74%) y, a nivel nacional, es del 32% frente al objetivo del 65% establecido por la Estrategia en Cáncer del Sistema Nacional de Salud^1^^,^^7^.

Los ejes que estructuran las desigualdades sociales en salud son los determinantes sociales de la salud^8^, que influyen en los estilos de vida y los patrones comportamentales de las personas^4^. En este sentido, diversos estudios han evidenciado que el sexo, la edad, los ingresos, el nivel de estudios, el país de origen o la existencia de programas de cribado poblacional, se relacionan con la participación en cribados en Europa^9^^-^^11^ y España^12^^-^^16^. Más aún, los determinantes sociales de la salud se interrelacionan y afectan de forma diferenciada a la salud de las mujeres^17^^-^^19^. Es por ello que resulta conveniente analizar su influencia en la participación en prácticas preventivas y cribados de cáncer desde la perspectiva de género.

En España existen dos fuentes de información sobre pruebas de detección precoz: las encuestas poblacionales de salud, con datos autodeclarados y de acceso público, y los registros de cribados poblacionales de cada comunidad, heterogéneos y de acceso limitado. La Encuesta Nacional de Salud (ENS) y la Encuesta Europea de Salud en España (EESE) son encuestas periódicas elaboradas por el Instituto Nacional de Estadística (INE) y el Ministerio de Sanidad. La EESE se diseña y lleva a cabo en consonancia con el resto de países europeos^20^. Recientemente se han unificado ambas encuestas en la Encuesta de Salud de España (ESdE) 2023^21^.

Si bien la EESE y la ENS han sido utilizadas en la literatura para analizar la participación en pruebas de cribado de CCR^12^^-^^16^, no se tiene constancia de estudios previos que integren la perspectiva de género y determinantes sociales de la salud, el análisis de tendencias temporales y los datos de la ESdE 2023.

El objetivo de este estudio es describir la evolución temporal de la realización de la prueba de detección precoz de cáncer colorrectal desde 2009 hasta 2023 en España, analizando las diferencias por comunidades autónomas, sexo y variables relativas a los determinantes sociales de la salud.

## MATERIAL Y MÉTODOS

*Diseño*. Estudio observacional transversal a partir de la Encuesta Europea de Salud en España 2009, 2014 y 2020, y de la Encuesta de Salud de España 2023. El diseño muestral y la recogida de información se describen en la metodología de cada encuesta^20^^-^^23^. Los datos son de acceso público en la página web del INE^24^.

*Población y muestra.* Se incluyó a la población diana del cribado de CCR, hombres y mujeres de 50 a 69 años. Se analizó cada base de datos por separado, aplicándose factores de elevación centrados en la muestra, calculados a partir de los factores de elevación proporcionados por el INE.

*Variables.* La *variable de respuesta* del estudio (variable dependiente) fue la realización de la prueba de cribado de CCR mediante la detección de sangre oculta en heces (SOH) que, cumpliendo con las recomendaciones vigentes en España para cada periodo de observación^7^^,^^25^, implica la realización de una prueba de sangre oculta en heces en los 24 meses previos en personas de 50 a 69 años. Además, puesto que las pruebas de cribado están dirigidas a población asintomática^26^, se añadió como criterio de realización adecuada que el motivo fuese *por consejo de su médico de atención primaria o especialista, aunque no tenía ningún problema* o *porque recibió una carta, le llamaron por teléfono o le dijeron en su centro de salud si se quería hacer esta prueba*, cuando estaba disponible dicha información (EESE 2014).

Se recogieron las siguientes *variables explicativas*:

- Sociodemográficas: comunidad autónoma (CCAA), existencia de cribado poblacional sistemático (2009 y 2014), tamaño del municipio de residencia (rural/urbano)^27^, edad agrupada en intervalos de 5 años^28^, país de nacimiento y nacionalidad, nivel de estudios, situación laboral, nivel de ingresos (según el salario mediano anual) y socioeconómico (según clase social ocupacional)^29^. La variable sexo se consideró como dicotómica (hombre, mujer) según sexo biológico, ya que era la información disponible en las encuestas.
- Entorno social y urbano: estado civil (persona soltera, casada, viuda, separada o divorciada), convivencia, tipo de hogar, grado de apoyo social (para todos los años: *En caso de problema personal grave, ¿con cuántas personas podría contar?* y, de 2014 a 2023, mediante la escala *Oslo Social Support* / OSS-3^30^), provisión de cuidados a otras personas, percepción de prestaciones sociales, y exposición a problemas en la vivienda.
- Estilo de vida: actividad física en el tiempo libre (en 2009, se obtuvo a partir del *International Physical Activity Questionnaire*^31^; en los demás años, se obtuvo de la respuesta a la pregunta *Frecuencia con la que realiza alguna actividad física en su tiempo libre*^32^) y *actividad física suficiente en total* (calculada para 2014-2023^33^). Otras variables fueron: alimentación saludable (consumo diario de frutas y verduras), consumo de tabaco y consumo de alcohol (habitual, con frecuencia semanal; ocasional, con frecuencia mensual; nunca o exconsumo).
- Estado de salud y sistema sanitario: salud autopercibida dicotomizada (positiva: muy buena, buena; negativa: regular, mala, muy mala); padecimiento de alguna enfermedad crónica (autorreportada) y limitaciones diarias (sí: gravemente limitada o limitada, pero no gravemente; no: nada limitada). Se incluyó la severidad en problemas de salud mental que, en 2009, se calculó a partir de la disfunción psicológica derivada del *Mental Health Index (MHI-5)* del *Short Form-36 Health Survey*^23^. En 2014-2023 se utilizó la variable “Severidad de la sintomatología depresiva” proporcionada en las encuestas. Asimismo, se incluyeron la cobertura sanitaria y la percepción de barreras en la asistencia médica.

### Análisis estadístico

Para analizar la prevalencia de realización adecuada de la prueba de SOH por CCAA y sexo, se calcularon frecuencias absolutas, porcentajes e intervalos de confianza al 95% (IC95%). Se utilizó la prueba Chi-cuadrado para las comparaciones dentro de cada grupo (hombres, mujeres). Además, se realizó un análisis *post hoc* mediante residuos estandarizados, aplicando la corrección de Bonferroni, para identificar las comunidades con diferencias significativas respecto a la realización esperada de la prueba bajo la hipótesis de no existencia de diferencias entre comunidades. Con el fin de identificar los factores asociados a la realización adecuada de SOH por sexo, se elaboraron tablas de contingencia para hombres y mujeres por variables explicativas (frecuencias absolutas, porcentajes e IC95%) y se aplicó la prueba Chi-cuadrado. El nivel de significación estadística se estableció en p<0,05 para todas las comparaciones. Se utilizó el programa IBM SPSS Statistics versión 29.0.1.0.

*Análisis de tendencias.* Para estudiar la evolución temporal de la realización de la prueba de SOH como cribado de CCR entre 2009 y 2023, identificando tendencias y puntos de cambio significativos (*joinpoint*), se empleó el análisis de regresión *joinpoint*. Se tomaron como base las CCAA en las que había programa de cribado implantado en el año de encuesta. Para cada segmento temporal se calcularon el porcentaje anual de cambio (APC) y su IC95%, aplicando una transformación logarítmica. El modelo fue estimado utilizando el criterio de información bayesiano (BIC) ponderado, asumiendo heterocedasticidad y no correlación en el cálculo de los errores estándar. Se utilizó el *Joinpoint Regression Program*, versión 5.4.0.0^34^.

## RESULTADOS

La muestra con información válida para la variable respuesta respecto de la población diana en cada año fue 5.684 personas en 2009 (98,68%), 6.239 en 2014 (95,38%), 6.742 en 2020 (98,78%) y 6.461 en 2023 (97,03%).

En general, la realización de la prueba de SOH en el total nacional aumentó desde 2009 hasta 2023 (3,4% en 2009, 9,2% en 2014, 31,9% en 2020 y 38,7% en 2023).

*Realización de la prueba por comunidades autónomas*. Las diferencias por CCAA fueron significativas en todos los años excepto en 2009 (Tabla 1). En 2014 se identificó un descenso en algunas CCAA mientras que las que ya tenían cribado implantado aumentaron la realización (Canarias, Cantabria, Castilla y León, Cataluña, Comunidad Valenciana, Galicia, Murcia, Navarra, País Vasco y La Rioja). Fue a partir de 2020 cuando se manifestó un marcado aumento generalizado, que se mantuvo en 2023 (excepto en Cantabria, Comunidad Valenciana, Navarra y País Vasco). Las comunidades que incrementaron el porcentaje de realización de forma constante de 2009 a 2023 fueron Castilla-La Mancha, Cataluña, Galicia, Murcia y La Rioja. Las ciudades autónomas Ceuta y Melilla mantuvieron porcentajes de realización marginales en todos los periodos.

**Tabla 1:** Realización adecuada de la prueba de detección precoz de cáncer colorrectal por comunidad autónoma, año de encuesta y sexo

Comunidad autónoma	2009		2014		2020		2023	
	Hombres	Mujeres	Hombres	Mujeres	Hombres	Mujeres	Hombres	Mujeres
	n (%)	n (%)	n (%)	n (%)	n (%)	n (%)	n (%)	n (%)
	IC95%	IC95%	IC95%	IC95%	IC95%	IC95%	IC95%	IC95%
p *(X^2^)*	0,073	0,615	<0,001	<0,001	<0,001	<0,001	<0,001	<0,001
Andalucía	18 (3,9)	6 (1,3)	14 (2,6)	6 (1,1)	100 (17,4)	71 (11,5)	157 (29,1)	157 (26,8)
	2,1-5,6	0,3-2,3	1,3-3,9	0,2-1,9	14,3-20,5	9,0-14,0	25,2-32,9	23,2-30,4
Aragón	3 (3,8)	2 (2,2)	3 (3,3)	2 (2,1)	20 (22,5)	27 (26,2)	43 (45,7)	40 (41,7)
	0-7,9)	0-5,1	0-6,9	0-5,0	13,8-31,1	17,7-34,7	35,7-55,8	31,8-51,5
Asturias	2 (2,9)	1 (1,2)	1 (1,2)	0	24 (29,6)	30 (33,7)	33 (42,9)	31 (40,3)
	0-6,8	0-3,6	0-3,6	-	19,7-3-9,6	23,9-43,5	31,8-53,9	29,3-51,2
Baleares	4 (6,1)	3 (4,8)	1 (1,5)	2 (2,7)	8 (9,1)	5 (6,7)	21 (27,3)	27 (37,5)
	0,3-11,8	0-10,2	0-4,4	0-6,5	3,1-15,1	1,0-12,3	17,3-37,2	26,3-48,7
Canarias	8 (6,3)	4 (3,0)	7 (5,2)	12 (9,1)	52 (28,6)	58 (33,9)	93 (50,3)	75 (46,0)
	2,1-10,5	0,1-6,0	1,4-8,9	4,2-14,0	22,0-35,1	26,8-41,0	43,1-57,5	38,4-53,7
Cantabria	1 (2,5)	1 (2,8)	13 (30,2)	10 (23,8)	15 (36,6)	20 (42,6)	9 (24,3)	11 (26,2)
	0-7,3	0-8,1	16,5-44,0	10,9-36,7	21,8-51,3	28,4-56,7	10,5-38,1	12,9-39,5
Castilla y León	4 (2,3)	5 (3,0)	9 (4,8)	8 (4,3)	89 (48,9)	79 (44,4)	77 (43,0)	92 (50,8)
	0,1-4,6	0,4-5,5	1,8-7,9	1,4-7,3	41,6-56,2	37,1-51,7	35,8-50,3	43,5-58,1
Castilla - La Mancha	3 (2,6)	1 (0,8)	4 (3,1)	1 (0,8)	31 (22,0)	46 (31,3)	66 (48,2)	50 (38,5)
	0-5,5	0-2,5	0,1-6,2	0-2,3	15,1-28,8	23,8-38,8	39,8-56,5	30,1-46,8
Cataluña	15 (3,3)	9 (1,9)	48 (10,1)	40 (7,7)	167 (32,3)	191 (36,0)	225 (43,9)	213 (43,4)
	1,7-4,9	0,7-3,1	7,4-12,8	5,4-10,0	28,3-36,3	32,0-40,1	39,6-48,2	39,0-47,8
Comunidad Valenciana	25 (8,3)	12 (4,1)	42 (13,2)	57 (15,8)	151 (40,8)	177 (49,2)	109 (32,6)	113 (29,2)
	5,2-11,5	1,8-6,4	9,5-16,9	12,1-19,6	35,8-45,8	44,0-54,3	27,6-37,7	24,7-33,7
Extremadura	3 (4,8)	1 (1,4)	2 (2,8)	2 (2,7)	11 (13,3)	5 (6,6)	16 (20,0)	17 (21,8)
	0-10,0	0-4,1	0-6,6	0-6,3	6,0-20,5	1,0-12,2	11,2-28,8	12,6-31,0
Galicia	4 (2,4)	5 (2,5)	14 (7,7)	8 (4,2)	82 (42,1)	96 (49,5)	106 (57,0)	103 (55,4)
	0,1-4,7	0,3-4,7	3,8-11,5	1,3-7,0	35,1-49,0	42,4-56,5	49,9-64,1	48,2-62,5
Madrid	15 (4,2)	11 (2,6)	8 (2,1)	8 (1,8)	123 (27,9)	126 (25,4)	135 (34,9)	155 (31,9)
	2,1-6,2	1,1-4,1	0,7-3,5	0,6-3,1	23,7-32,1	21,5-29,2	30,1-39,6	27,7-36,0
Murcia	5 (7,2)	2 (2,9)	17 (19,8)	19 (21,8)	34 (33,3)	29 (30,2)	50 (50,5)	47 (47,0)
	1,1-13,4	0-6,9	11,4-28,2	13,2-30,5	24,2-42,5	21,0-39,4	40,7-60,4	37,2-56,8
Navarra	0	1 (2,3)	3 (6,8)	5 (11,4)	29 (67,4)	33 (68,8)	31 (64,6)	28 (63,6)
	-	0-6,8	0-14,3	2,0-20,7	53,4-81,4	55,6-81,9	51,1-78,1	49,4-77,9
País Vasco	11 (7,6)	8 (5,0)	92 (57,5)	102 (60,0)	103 (66,0)	108 (64,7)	80 (55,2)	73 (46,5)
	3,3-11,9	1,6-8,3	49,8-65,2	52,6-67,4	58,6-73,5	57,4-71,9	47,1-63,3	38,7-54,3
Rioja	0	0	6 (27,3)	6 (28,6)	6 (30,0)	7 (30,4)	7 (30,4)	9 (34,6)
	-	-	8,7-45,9	9,2-47,9	9,9-50,1	11,6-49,2	11,6-49,2	16,3-52,9
Ceuta	0	0	0	0	0	0	0	0
	-	-	-	-	-	-	-	-
Melilla	0	0	0	0	0	0	1 (16,7)	1 (20,0)
	-	-	-	-	-	-	0-46,5	0-55,1
España	121 (4,4)	72 (2,5)	284 (9,4)	288 (8,9)	1045 (31,5)	1108 (32,3)	1259 (40,0)	1242 (37,5)
	3,6-5,1	1,9-3,0	8,3-10,4	8,0-9,9	29,9-33,1	30,8-33,9	38,2-41,7	35,9-39,2

IC95%: intervalo de confianza al 95%. El análisis *post hoc* de la prueba Chi-cuadrado, comparando mediante residuos estandarizados comunidades autónomas dentro de cada sexo, se realizó aplicando la corrección de Bonferroni. En amarillo, comunidades con exceso significativo de realización; en verde, comunidades con déficit significativo de realización.

En 2009, en la mayoría de CCAA se realizaron la prueba mas hombres que mujeres. Este patrón empezó a cambiar en 2014 en al menos siete CCAA, de entre las que destacaron Comunidad Valenciana, Murcia, País Vasco o La Rioja por exceso significativo de realización en mujeres respecto de lo esperado.

**Figura 1:**
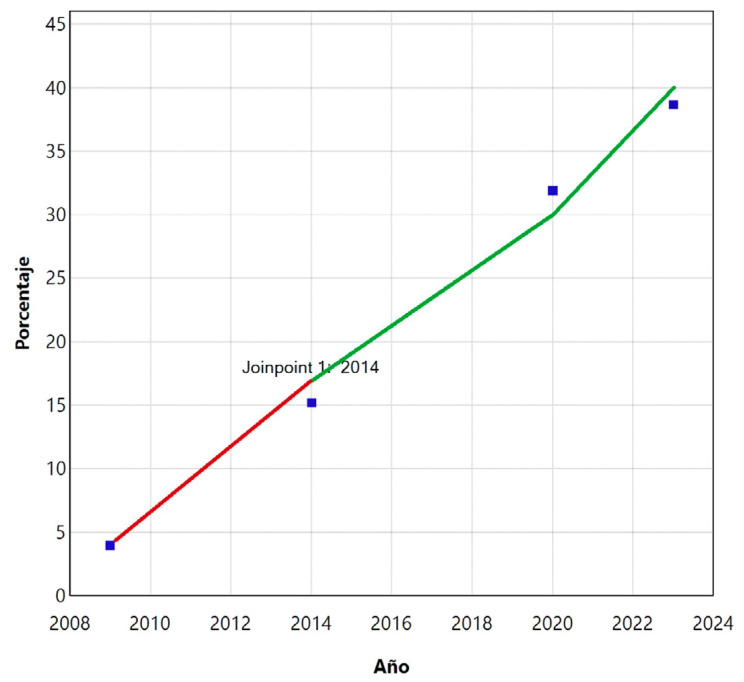
Realización de la prueba de sangre oculta en heces (%) entre la población de las comunidades autónomas con programa de cribado en 2009-2023. En azul, los puntos de cambio (*joinpoints*) observados, en 2009-2014 (rojo) y en 2014-2023 (verde).

*Tendencias temporales.* Si bien la tendencia global fue creciente, los resultados de la regresión *joinpoint* encontraron dos tramos diferenciados, situando el *joinpoint* en 2014 (Fig. 1). El APC para el primer tramo 2009-2014 fue mayor que para 2014-2023, ambos significativos (p<0,000001). A partir de 2014 cae el APC a una tercera parte: de 33,56 (IC95%: 13,23-77,06) a 10,02 (IC95%: 4,57-15,75). Según el modelo de regresión *joinpoint* obtenido, si se mantuviese el mismo APC del 10%, se llegaría a un 64,4% de participación en 2028.

*Diferencias en la realización de la prueba de sangre oculta en heces por variables explicativas, sexo y año de encuesta.* En términos generales, la distribución porcentual entre las categorías de las variables se mantuvo a lo largo del tiempo, si bien los valores aumentaron con el aumento de personas que se realizaron la prueba en total. Las diferencias por sexo no fueron significativas en el total de España en 2014 y 2020, pero sí en 2009 y 2023 (Tabla 2).

La realización de la prueba fue mayor en CCAA con cribado (diferencias significativas en 2014), en proporción similar para ambos sexos. A lo largo de los años se manifestó un gradiente de edad en mujeres y hombres, con mayor realización de SOH en grupos de más edad (diferencias significativas de 2014 a 2023). El nivel de estudios, ingresos y socioeconómico presentaron diferencias significativas en todos los años -excepto para las mujeres en 2009-, con mayor realización de SOH en niveles altos. Sobresalen algunas diferencias por sexo en la situación laboral puesto que, excepto en 2009, las personas que más se realizaron la prueba fueron las mujeres que tenían incapacidad para trabajar y los hombres que estaban jubilados.

**Tabla 2:** Diferencias en la distribución de la realización adecuada de la prueba de detección de cáncer colorrectal por variables sociodemográficas, sexo y año de encuesta

Variable	2009		2014		2020		2023	
	Hombres	Mujeres	Hombres	Mujeres	Hombres	Mujeres	Hombres	Mujeres
	n (%)	n (%)	n (%)	n (%)	n (%)	n (%)	n (%)	n (%)
	IC95%	IC95%	IC95%	IC95%	IC95%	IC95%	IC95%	IC95%
*Sexo*								
	p<0,001		p=0,624		p=0,488		p=0,045	
	122 (4,4)	71 (2,4)	281 (9,3)	288 (8,9)	1045 (31,5)	1107 (32,3)	1259 (40,0)	1242 (37,5)
	3,6-5,2	1,9-3,0	8,3-10,3	8,0-9,9	29,9-33,1	30,7-33,9	38,2-41,7	35,9-39,2
Cribado poblacional^*^								
	p=0,119	p=0,447	p<0,001	p<0,001				
Sí	46 (5,3)	24 (2,8)	250 (15,2)	268 (15,3)				
	3,8-6,8	1,7-3,9	13,4-16,9	13,6-17,0				
No	76 (4,0)	47 (2,3)	32 (2,3)	21 (1,4)				
	3,1-4,9	1,6-2,9	1,5-3,1	0,8-2,0				
*Edad*								
	p=0,712	p=0,090	p=0,007	p=0,004	p<0,001	p<0,001	0,052	<0,001
50-54	39 (4,6)	13 (1,5)	64 (6,7)	60 (6,3)	231 (22,0)	245 (24,5)	332 (36,2)	257 (27,8)
	3,2-6,0	0,7-2,3	5,1-8,3	4,8-7,9	19,5-24,5	21,8-27,1	33,1-39,3	25,0-30,7
55-59	36 (5,0)	25 (3,4)	77 (9,6)	88 (10,5)	267 (31,4)	299 (32,1)	350 (41,9)	394 (42,9)
	3,4-6,6	2,1-4,7	7,6-11,7	8,4-12,6	28,3-34,5	29,1-35,1	38,5-45,2	39,7-46,1
60-64	24 (3,7)	20 (2,8)	75 (11,1)	76 (10,8)	311 (38,4)	314 (38,6)	329 (41,4)	323 (40,8)
	2,2-5,2	1,6-3,9	8,7-13,5	8,5-13,1	35,1-41,8	35,3-42,0	38,0-44,8	37,4-44,2
65-69	24 (4,4)	13 (2,1)	65 (11,0)	64 (8,7)	236 (38,9)	249 (36,7)	248 (41,2)	269 (39,7)
	2,7-6,2	1,0-3,3	8,5-13,5	6,7-10,8	35,0-42,8	33,0-40,3	37,3-45,1	36,0-43,4
*Tamaño del municipio de residencia*								
	p=0,982	p=0,537	p=0,047	p=0,138	p=0,829	p=0,009	p=0,475	p=0,062
Rural	28 (4,4)	12 (2,1)	52 (7,4)	48 (7,5)	242 (31,8)	228 (36,7)	283 (38,8)	267,0 (40,7)
	2,8-6,0	0,9-3,2	5,5-9,3	5,4-9,5	28,5-35,2	32,9-40,5	35,3-42,4	36,9-44,5
Urbano	94 (4,4)	59 (2,5)	229 (9,9)	240 (9,3)	803 (31,4)	879 (31,3)	976 (40,3)	976,0 (36,8)
	3,5-5,3	1,9-3,2	8,7-11,1	8,2-10,4	29,6-33,2	29,6-33,0	38,3-42,3	34,9-38,6
*País de nacimiento*								
	p=0,237	p=0,087	p=0,002	p=0,246	p<0,001	p=0,024	p<0,001	p<0,001
España	110 (4,3)	70 (2,6)	273 (9,8)	268 (9,1)	978 (33,1)	1003 (33,0)	1121 (41,2)	1116 (39,4)
	3,5-5,1	2,0-3,2	8,7-10,9	8,1-10,2	31,4-34,8	31,3-34,6	39,4-43,1	37,6-41,1
Extranjero	13 (6,0)	1 (0,5)	8 (3,5)	20 (7,1)	67 (18,5)	104 (27,1)	138 (31,9)	126 (26,6)
	2,9-9,2	0-1,5	1,1-5,9	4,1-10,1	14,5-22,4	22,6-31,5	7,5-36,3	22,7-30,6
*Nacionalidad española*								
	p=0,097	p=0,261	p=0,017	p=0,294	p<0,001	p=0,034	p<0,001	p<0,001
Sí	111 (4,3)	70 (2,5)	275 (9,6)	275 (9,1)	1009 (32,9)	1044 (32,8)	1191 (41,4)	1169 (39,3)
	3,5-5,1	1,9-3,1	8,5-10,7	8,1-10,1	31,2-34,5	31,1-34,4	39,6-43,2	37,6-41,1
No	12 (7,0)	1 (0,7)	6 (3,9)	14 (6,9)	36 (14,7)	63 (26,1)	68 (24,8)	74 (21,9)
	3,2-10,8	0-2,1	0,8-6,9	3,4-10,4	10,3-19,1	20,6-31,7	19,7-29,9	17,5-26,3
*Nivel de estudios*								
	p=0,011	p=0,868	p<0,001	p=0,050	p=0,026	p<0,001	p<0,001	p<0,001
Sin estudios	16 (4,0)	13 (2,2)	16 (6,5)	20 (5,3)	42 (21,9)	53 (25,5)	6 (66,7)	2 (15,4)
	2,1-5,9	1,0-3,3	3,4-9,6	3,0-7,6	16,0-27,7	19,6-31,4	35,9-97,5	0-35,0
Primarios	39 (3,2)	34 (2,5)	112 (7,6)	152 (9,1)	476 (31,5)	442 (29,4)	451 (34,3)	466 (33,6)
	2,2-4,2	1,6-3,3	6,2-8,9	7,7-10,5	29,2-33,9	27,1-31,7	31,7-36,9	31,1-36,1
Secundarios	30 (5,5)	12 (2,3)	63 (10,8)	53 (9,8)	220 (32,6)	289 (37,8)	330 (45,0)	329 (42,5)
	3,6-7,4	1,0-3,5	8,3-13,4	7,3-12,3	29,1-36,1	34,4-41,3	41,4-48,6	39,0-45,9
Superiores	38 (6,4)	12 (3,0)	91 (12,7)	64 (10,2)	307 (32,7)	322 (33,9)	438 (43,9)	419 (41,4)
	4,4-8,3	1,3-4,6	10,3-15,2	7,8-12,6	29,7-35,7	30,9-36,9	40,9-47,0	38,3-44,4
*Situación laboral*								
	p=0,150	p=0,430	p=0,005	p=0,838	p<0,001	p<0,001	p=0,047	p=0,384
Trabajando	74 (5,1)	22 (2,2)	139 (9,3)	113 (9,8)	581 (30,2)	499 (31,1)	750 (39,5)	592 (36,5)
	4,0-6,2	1,3-3,2	7,9-10,8	8,1-11,5	28,2-32,3	28,9-33,4	37,3-41,7	34,1-38,8
Desempleo	4 (1,4)	5 (2,5)	29 (6,7)	29 (9,0)	73 (20,3)	94 (29,1)	118 (36,4)	163 (38,4)
	0,0-2,7	0,3-4,6	4,4-9,1	5,9-12,1	16,2-24,5	24,1-34,1	31,2-41,7	33,7-43,0
Estudiando	0	0	0	0	0	0	0	0
	-	-	-	-	-	-	-	-
Jubilación	34 (4,2)	13 (2,3)	107 (12,2)	64 (9,0)	332 (38,6)	249 (37,7)	302 (40,3)	268 (39,5)
	2,8-5,6	1,1-3,6	10,0-14,4	6,9-11,1	35,4-41,9	34,0-41,4	36,8-43,8	35,8-43,2
Incapacidad	8 (4,7)	6 (5,6)	6 (3,6)	8 (7,4)	55 (34,4)	43 (43,9)	82 (50,3)	57 (44,5)
	1,5-7,8	1,2-10,0	0,8-6,5	2,5-12,3	27,0-41,7	34,1-53,7	42,6-58,0	35,9-53,1
Labores del hogar	1 (5,0)	24 (2,3)	0	74 (8,1)	1 (14,3)	219 (29,9)	3 (50,0)	162 (35,8)
	0-14,6	1,4-3,2	-	6,3-9,9	0-40,2	26,6-33,2	10,0-90,0	31,4-40,3
*Nivel socioeconómico*								
			p<0,001	p<0,001	p=0,010	p<0,001	p<0,001	p<0,001
Alto			72 (11,8)	70 (11,4)	212 (33,9)	262 (37,0)	303 (46,0)	285 (40,4)
			9,2-14,4	8,9-13,9	30,2-37,6	33,4-40,6	42,2-49,9	36,8-44,0
Medio			122 (11,2)	116 (10,4)	382 (34,0)	378 (34,3)	479 (42,4)	441 (42,8)
			9,3-13,0	8,6-12,2	31,2-36,7	31,5-37,1	39,5-45,3	39,8-45,8
Bajo			88 (6,8)	95 (6,7)	435 (29,0)	437 (29,4)	466 (35,1)	473 (33,1)
			5,4-8,1	5,4-8,0	26,7-31,3	27,1-31,7	32,5-37,7	30,7-35,6
*Nivel de ingresos*								
	p=0,019	p=0,620	p<0,001	p=0,001	p<0,001	p<0,001	p<0,001	p<0,001
Alto	57 (6,7)	18 (2,4)	145 (11,0)	135 (10,5)	714 (34,3)	722 (34,1)	1001 (42,0)	970 (39,2)
	5,0-8,4	1,3-3,5	9,3-12,7	8,9-12,2	32,3-36,4	32,1-36,2	40,0-44,0	37,3-41,1
Bajo	19 (3,7)	14 (2,0)	78 (7,0)	89 (7,0)	320 (26,2)	364 (28,3)	209 (32,0)	225 (31,0)
	2,1-5,3	1,0-3,1	5,5-8,5	5,6-8,4	23,7-28,6	25,8-30,7	28,4-35,6	27,7-34,4
*Estado civil*								
	p=0,568	p=0,167	p=0,206	p=0,667	p<0,001	p=0,423	p=0,475	p<0,001
Soltera	9 (3,7)	5 (2,1)	22 (6,5)	24 (9,1)	94 (22,9)	96 (29,9)	181 (37,9)	128 (33,8)
	1,3-6,1	0,3-3,9	3,9-9,2	5,6-12,5	18,8-26,9	24,9-34,9	33,5-42,2	29,0-38,5
Casada	103 (4,4)	50 (2,4)	239 (9,8)	215 (9,3)	867 (33,7)	814 (33,2)	934 (40,6)	907 (40,7)
	3,6-5,3	1,7-3,0	8,7-11,0	8,1-10,5	31,9-35,5	31,3-35,0	38,6-42,6	38,7-42,7
Viuda	2 (3,0)	7 (1,9)	6 (9,1)	26 (7,6)	17 (27,9)	78 (31,8)	17 (32,7)	57 (28,2)
	0-7,2	0,5-3,3	2,2-16,0	4,8-10,4	16,6-39,1	26,0-37,7	19,9-45,4	22,0-34,4
Separada/divorciada	9 (6,5)	10 (4,7)	14 (7,6)	23 (7,8)	66 (25,3)	117 (29,8)	120 (39,0)	143 (30,1)
	2,4-10,6	1,9-7,5	3,8-11,4	4,7-10,8	20,0-30,6	25,3-34,4	33,5-44,4	26,0-34,2

*: se diferencia por comunidades autónomas con y sin cribado poblacional implantado para los años de encuesta 2009 y 2014, a partir de 2020 todas tienen implantado el cribado poblacional; IC95%: intervalo de confianza al 95%. Valores de p obtenidos mediante la prueba de Chi-cuadrado, excepto en caso de frecuencias esperadas pequeñas, que se aplicó la prueba exacta de Fisher. Los espacios en blanco sin datos indican que dicha información no estaba disponible para el año de encuesta.

Quienes convivían con alguien se realizaron la prueba en mayor proporción, con diferencias significativas para ambos sexos en 2023 (Tabla 3). Ocurrió lo mismo con las personas que vivían solo con su pareja, en contraste con quienes vivían en soledad o con descendencia, que se realizaron la SOH en menor porcentaje en todos los años y para ambos sexos. Hubo menor realización de la prueba entre quienes percibían un grado de apoyo social bajo. Quienes cuidaban de alguien se realizaron la prueba en mayor proporción. Los problemas en la vivienda se relacionaron con una mayor realización de SOH de forma significativa en ambos sexos, especialmente en 2023.

**Tabla 3:** Diferencias en la distribución de la realización adecuada de la prueba de detección de cáncer colorrectal por variables del entorno social y urbano, sexo y año de encuesta

Variable	2009		2014		2020		2023	
	Hombres	Mujeres	Hombres	Mujeres	Hombres	Mujeres	Hombres	Mujeres
	n (%)	n (%)	n (%)	n (%)	n (%)	n (%)	n (%)	n (%)
	IC95%	IC95%	IC95%	IC95%	IC95%	IC95%	IC95%	IC95%
*Convivencia*								
	p=0,187	p=0,474	p=0,084	p=0,264	p<0,001	p=0,323	p=0,017	p<0,001
Sí	109 (4,6)	49 (2,3)	238 (9,8)	213 (9,3)	847 (33,5)	794 (32,9)	933 (41,1)	893 (41,0)
	3,8-5,5	1,7-3,0	8,6-11,0	8,1-10,5	31,7-35,4	31,0-34,7	39,1-43,1	38,9-43,1
No	13 (3,2)	22 (2,8)	43 (7,5)	74 (8,1)	197 (25,5)	307 (31,1)	307 (36,4)	337 (31,0)
	1,5-4,9	1,6-3,9	5,3-9,6	6,3-9,8	22,4-28,6	28,2-34,0	33,2-39,7	28,3-33,8
*Tipo de hogar*								
	p=0,035	p=0,293	p=0,106	p=0,194	p<0,001	p<0,001	p=0,010	p<0,001
Unipersonal	9 (3,9)	13 (3,9)	21 (6,3)	28 (7,2)	104 (27,5)	123 (31,4)	183 (35,7)	188 (37,2)
	1,4-6,4	1,8-5,9	3,7-8,9	4,7-9,8	23,0-32,0	26,8-36,0	31,5-39,8	33,0-41,4
Pareja	56 (5,8)	22 (2,3)	100 (10,6)	105 (10,6)	351 (37,7)	355 (38,7)	405 (44,4)	404 (41,9)
	4,3-7,3	1,3-3,2	8,6-12,5	8,7-12,5	34,6-40,8	35,6-41,9	41,1-47,6	38,8-45,0
Pareja con hijos	39 (3,4)	24 (2,5)	131 (9,7)	92 (8,3)	448 (31,2)	369 (29,1)	466 (38,1)	437 (39,7)
	2,3-4,4	1,5-3,5	8,2-11,3	6,7-9,9	28,8-33,6	26,6-31,6	35,4-40,9	36,8-42,6
Un progenitor con hijos	2 (1,7)	7 (2,4)	10 (7,3)	27 (7,7)	54 (23,3)	109 (28,9)	90 (39,5)	118 (28,7)
	0-4,0	0,6-4,1	2,9-11,7	4,9-10,5	17,8-28,7	24,3-33,5	33,1-45,8	24,3-33,1
Otro	16 (5,4)	5 (1,3)	19 (7,3)	36 (9,4)	89 (26,3)	151 (32,1)	115 (41,7)	96 (29,2)
	2,8-8,0	0,2-2,5	4,1-10,5	6,5-12,4	21,6-31,0	27,9-36,3	35,9-47,5	24,3-34,1
*Apoyo social*								
	p=0,412	p=0,996	p=0,504	p=0,837	p=0,001	p=0,372	p=0,902	p=0,926
Bajo	1 (2,2)	1 (2,6)	2 (5,4)	3 (6,5)	3 (9,4)	9 (22,5)	15 (36,6)	10 (40,0)
	0-6,5	0-7,5	0-12,7	0-13,7	0-19,5	9,6-35,4	21,8-51,3	20,8-59,2
Medio	28 (5,4)	14 (2,3)	54 (8,6)	61 (8,9)	170 (27,6)	209 (31,8)	303 (40,1)	256 (38,0)
	3,4-7,3	1,1-3,6	6,4-10,7	6,8-11,0	24,0-31,1	28,2-35,4	36,6-43,6	34,3-41,6
Alto	93 (4,3)	54 (2,4)	225 (9,6)	223 (9,0)	867 (32,7)	888 (32,7)	924 (40,1)	961 (37,4)
	3,4-5,1	1,8-3,0	8,4-10,8	7,9-10,2	30,9-34,5	30,9-34,4	38,1-42,1	35,5-39,2
*Apoyo social (OSS-3)*								
			p=0,295	p=0,979	p<0,001	p=0,904	p=0,070	p=0,010
Pobre			7 (6,2)	13 (8,4)	17 (18,7)	29 (30,2)	62 (34,4)	68 (38,9)
			1,8-10,6	4,1-12,8	10,7-26,7	21,0-39,4	27,5-41,4	31,6-46,1
Moderado			105 (10,2)	95 (8,8)	283 (26,7)	336 (32,4)	465 (38,4)	426 (34,4)
			8,4-12,1	7,1-10,5	24,1-29,4	29,6-35,2	35,7-41,1	31,7-37,0
Fuerte			163 (9,1)	170 (8,9)	723 (34,6)	724 (32,4)	684 (41,6)	712 (39,8)
			7,7-10,4	7,6-10,2	32,6-36,7	30,4-34,3	39,2-44,0	37,5-42,0
*Cuidados (horas semanales)*								
			p=0,220	p=0,042	p=0,007	p=0,106	p<0,001	p<0,001
Sí, <10			20 (12,7)	25 (13,3)	75 (42,6)	78 (37,1)	97 (50,8)	113 (50,7)
			7,5-18,0	8,4-18,2	35,3-49,9	30,6-43,7	43,7-57,9	44,1-57,2
Sí, 10 a 20			5 (5,8)	16 (13,1)	27 (36,5)	68 (37,8)	57 (64,0)	44 (31,4)
			0,9-10,8	7,1-19,1	25,5-47,5	30,7-44,9	54,1-74,0	23,7-39,1
Sí, ≥20			18 (11,5)	38 (9,6)	53 (28,8)	121 (33,6)	93 (48,2)	125 (43,6)
			6,5-16,4	6,7-12,5	22,3-35,3	28,7-38,5	41,1-55,2	37,8-49,3
No			238 (9,1)	209 (8,4)	886 (30,8)	836 (31,3)	993 (37,6)	947 (36,1)
			8,0-10,2	7,3-9,4	29,1-32,5	29,6-33,1	35,7-39,4	34,2-37,9
*Prestaciones sociales*								
	p=0,083	p=0,979	p=0,290	p=0,428	p=0,217	p<0,001	p=0,121	p=0,653
Sí	55 (3,8)	44 (2,4)	161 (9,9)	194 (9,3)	517 (32,7)	656 (35,3)	601 (41,4)	629 (38,0)
	2,8-4,7	1,7-3,1	8,4-11,3	8,0-10,5	30,4-35,0	33,1-37,5	38,9-43,9	35,7-40,4
No	66 (5,1)	26 (2,4)	121 (8,8)	94 (8,4)	528 (30,7)	448 (28,9)	652 (38,7)	606 (37,3)
	3,9-6,3	1,5-3,3	7,3-10,3	6,8-10,1	28,5-32,9	26,6-31,2	36,3-41,0	34,9-39,6
*Ruido*								
	p=0,020	p=0,809					p<0,001	p=0,002
Sí	44 (5,9)	22 (2,5)					420 (45,4)	398 (41,6)
	4,2-7,6	1,5-3,6					42,1-48,6	38,5-44,8
No	78 (3,9)	49 (2,4)					833 (37,7)	841 (35,8)
	3,0-4,7	1,7-3,0					35,6-39,7	33,9-37,7
*Contaminación*								
	p=0,002	p=0,057					p=0,001	p<0,001
Sí	37 (7,0)	24 (3,4)					346 (44,8)	361 (44,1)
	4,8-9,1	2,1-4,8					41,3-48,3	40,7-47,5
No	85 (3,8)	47 (2,1)					888 (38,2)	845 (35,0)
	3,0-4,6	1,5-2,7					36,3-40,2	33,1-36,9
*Malos olores*								
	p=0,156	p=0,146					p=0,497	p=0,038
Sí	25 (5,7)	19 (3,3)					225 (41,3)	263 (41,1)
	3,5-7,9	1,8-4,7					37,2-45,4	37,3-44,9
No	97 (4,2)	52 (2,2)					1031 (39,7)	975 (36,7)
	3,4-5,0	1,6-2,8					37,8-41,6	34,8-38,5
*Inseguridad*								
	p=0,037	p=0,501					p<0,001	p=0,270
Sí	32 (6,2)	16 (2,8)					387 (44,5)	372 (39,0)
	4,1-8,3	1,5-4,2					41,2-47,8	35,9-42,1
No	91 (4,1)	55 (2,3)					853 (37,9)	859 (37,0)
	3,3-4,9	1,7-3,0					35,9-39,9	35,0-38,9
*Mala calidad del agua*								
							p=0,005	p=0,001
Sí							373 (43,9)	406 (41,5)
							40,5-47,2	38,4-44,6
No							863 (38,4)	811 (35,5)
							36,4-40,4	33,5-37,5
*Poca limpieza de las calles*								
							p<0,001	p<0,001
Sí							532 (43,8)	587 (42,0)
							41,0-46,6	39,4-44,6
No							719 (37,5)	650 (34,2)
							35,3-39,7	32,0-36,3
*Escasez de zonas verdes*								
							p=0,757	p=0,055
Sí							360 (40,3)	389 (40,0)
							37,1-43,5	36,9-43,1
No							892 (39,7)	850 (36,4)
							37,7-41,7	34,5-38,4
*Molestias por animales*								
							p=0,024	p<0,001
Sí							441 (42,7)	520 (45,1)
							39,7-45,7	42,2-48,0
No							809 (38,5)	718 (33,5)
							36,4-40,6	31,5-35,5

IC95%: intervalo de confianza al 95%. Valores de p obtenidos mediante la prueba de Chi-cuadrado, excepto en caso de frecuencias esperadas pequeñas, que se aplicó la prueba exacta de Fisher. Los espacios en blanco sin datos indican que dicha información no estaba disponible para el año de encuesta.

Las variables de estilo de vida mostraron diferencias estadísticamente significativas a partir de 2014, con una mayor realización de SOH en hombres y mujeres activos, con alimentación saludable y no fumadores o exfumadores (Tabla 4). Las mujeres que consumieron alcohol de forma habitual se realizaron la prueba en mayor proporción.

**Tabla 4:** Diferencias en la distribución de la realización adecuada de la prueba de detección de cáncer colorrectal por variables del estilo de vida, sexo y año de encuesta

Variable	2009		2014		2020		2023	
	Hombres	Mujeres	Hombres	Mujeres	Hombres	Mujeres	Hombres	Mujeres
	n (%)	n (%)	n (%)	n (%)	n (%)	n (%)	n (%)	n (%)
	IC95%	IC95%	IC95%	IC95%	IC95%	IC95%	IC95%	IC95%
*Actividad física (tiempo libre)*								
	p=0,292	p=0,550	p<0,001	p=0,002	p<0,001	p<0,001	p=0,003	p<0,001
Activo	53 (5,0)	35 (2,6)	87 (13,7)	61 (12,6)	288 (37,1)	283 (43,0)	331 (44,4)	353 (46,4)
	3,7-6,3	1,8-3,5	11,0-16,4	9,6-15,6	33,7-40,5	39,2-46,8	40,9-48,0	42,8-49,9
Inactivo	70 (4,1)	36 (2,3)	194 (8,2)	227 (8,3)	757 (29,9)	823 (29,8)	903 (38,4)	881 (34,9)
	3,2-5,1	1,5-3,0	7,1-9,3	7,3-9,3	28,1-31,6	28,1-31,5	36,4-40,3	33,0-36,8
*Actividad física suficiente en total*								
			p<0,001	p<0,001	p<0,001	p<0,001	p<0,001	p<0,001
Sí			165 (11,3)	145 (11,1)	592 (34,2)	515 (36,8)	643 (42,5)	529 (42,4)
			9,7-13,0	9,4-12,8	31,9-36,4	34,2-39,3	40,0-45,0	39,6-45,1
No			115 (7,5)	141 (7,5)	449 (28,8)	583 (29,1)	553 (36,3)	658 (33,7)
			6,1-8,8	6,3-8,7	26,5-31,0	27,1-31,1	33,9-38,8	31,6-35,8
*Alimentación saludable*								
	p=0,552	p=0,799	p=0,113	p=0,031	p<0,001	p<0,001	p<0,001	p<0,001
Sí	76 (4,6)	47 (2,4)	122 (10,3)	156 (10,0)	427 (36,3)	620 (35,9)	357 (47,5)	495 (45,4)
	3,6-5,6	1,7-3,1	8,6-12,1	8,5-11,5	33,5-39,0	33,7-38,2	44,0-51,1	42,5-48,4
No	46 (4,1)	24 (2,5)	158 (8,6)	130 (7,9)	618 (28,9)	485 (28,5)	897 (37,5)	744 (33,6)
	3,0-5,3	1,5-3,5	7,3-9,9	6,6-9,1	27,0-30,8	26,4-30,7	35,6-39,4	31,7-35,6
*Tabaco*								
	p=0,088	p=0,019	p=0,017	p=0,005	p<0,001	p<0,001	p<0,001	p<0,001
Fumador	26 (3,1)	13 (2,6)	65 (7,2)	39 (5,8)	246 (27,0)	192 (26,2)	240 (31,0)	221 (35,6)
	1,9-4,3	1,2-4,0	5,5-8,8	4,0-7,6	24,1-29,9	23,0-29,4	27,7-34,2	31,9-39,4
Exfumador	55 (5,2)	19 (4,3)	130 (9,8)	76 (9,3)	443 (36,1)	341 (41,4)	491 (44,5)	387 (45,3)
	3,9-6,5	2,4-6,1	8,2-11,4	7,3-11,3	33,4-38,8	38,0-44,7	41,6-47,4	41,9-48,6
No fumador	36 (4,5)	38 (2,0)	86 (11,0)	173 (10,0)	355 (30,3)	571 (30,6)	526 (41,5)	632 (34,6)
	3,1-5,9	1,4-2,6	8,8-13,2	8,6-11,4	27,7-32,9	28,5-32,7	38,8-44,2	32,4-36,8
*Consumo alcohol*								
	p=0,371	p=0,120	p=0,019	p<0,001	p<0,001	p<0,001	p=0,150	p<0,001
Habitual	55 (4,1)	14 (2,8)	182 (9,8)	120 (12,6)	634 (34,2)	387 (39,6)	656 (40,8)	399 (43,8)
	3,0-5,1	1,3-4,2	8,5-11,2	10,5-14,7	32,0-36,3	36,5-42,7	38,4-43,2	40,5-47,0
Ocasional	37 (5,2)	28 (3,2)	63 (10,8)	80 (8,7)	230 (30,7)	347 (32,7)	355 (40,7)	431 (35,1)
	3,6-6,9	2,0-4,4	8,3-13,3	6,9-10,6	27,4-34,1	29,9-35,5	37,4-43,9	32,4-37,7
Nunca	24 (3,8)	28 (1,9)	37 (6,4)	88 (6,5)	181 (25,5)	373 (27,0)	235 (36,5)	400 (34,9)
	2,3-5,3	1,2-2,6	4,4-8,4	5,2-7,9	22,3-28,7	24,6-29,3	32,8-40,3	32,1-37,6

IC95%: intervalo de confianza al 95%. Valores de p obtenidos mediante la prueba de Chi-cuadrado, excepto en caso de frecuencias esperadas pequeñas, que se aplicó la prueba exacta de Fisher. Los espacios en blanco sin datos indican que dicha información no estaba disponible para el año de encuesta.

**Tabla 5:** Diferencias en la distribución de la realización adecuada de la prueba de detección de cáncer colorrectal por variables de estado de salud y sistema sanitario, sexo y año de encuesta

Variable	2009		2014		2020		2023	
	Hombres	Mujeres	Hombres	Mujeres	Hombres	Mujeres	Hombres	Mujeres
	n (%)	n (%)	n (%)	n (%)	n (%)	n (%)	n (%)	n (%)
	IC95%	IC95%	IC95%	IC95%	IC95%	IC95%	IC95%	IC95%
*Salud autopercibida*								
	p<0,001	p<0,001	p=0,778	p=0,003	p=0,001	p=0,185	p<0,001	p<0,001
Positiva	59 (3,3)	23 (1,4)	193 (9,4)	195 (10,2)	740 (30,0)	739 (31,6)	764 (35,8)	699 (33,4)
	2,5-4,1	0,8-2,0	8,1-10,7	8,9-11,6	28,2-31,8	29,7-33,5	33,8-37,8	31,4-35,4
Negativa	63 (6,5)	48 (3,8)	88 (9,1)	94 (7,2)	305 (36,1)	368 (33,9)	495 (48,7)	543 (44,7)
	4,9-8,0	2,7-4,8	7,3-10,9	5,8-8,6	32,8-39,3	31,0-36,7	45,6-51,7	41,9-47,4
*Enfermedad crónica*								
	p<0,001	p=0,014	p=0,697	p=0,119	p<0,001	p<0,001	p<0,001	p<0,001
Sí	93 (5,5)	59 (2,9)	195 (9,4)	233 (9,4)	785 (37,7)	836 (35,1)	931 (45,1)	1000 (42,4)
	4,4-6,6	2,2-3,6	8,2-10,7	8,2-10,5	35,6-39,7	33,2-37,1	42,9-47,2	40,4-44,3
No	30 (2,8)	12 (1,4)	86 (9,0)	55 (7,5)	260 (21,1)	271 (25,9)	312 (29,7)	231 (25,1)
	1,8-3,7	0,6-2,1	7,2-10,8	5,6-9,4	18,9-23,4	23,3-28,6	27,0-32,5	22,3-27,9
*Limitaciones diarias*								
	p=0,007	p<0,001	p=0,606	p=0,025	p=0,363	p=0,252	p<0,001	p<0,001
Sí	45 (6,2)	41 (4,0)	78 (9,8)	76 (7,3)	261 (32,8)	327 (33,8)	425 (46,2)	505 (47,8)
	4,4-7,9	2,8-5,2	7,7-11,8	5,7-8,9	29,6-36,1	30,8-36,8	43,0-49,5	44,8-50,8
No	77 (3,8)	30 (1,6)	203 (9,1)	212 (9,7)	784 (31,1)	780 (31,7)	827 (37,3)	727 (32,6)
	3,0-4,6	1,0-2,2	7,9-10,3	8,5-11,0	29,3-32,9	29,9-33,6	35,3-39,3	30,6-34,5
*Disfunción o severidad en problemas de salud mental*								
	p=0,238	p=0,067	p=0,475	p=0,118	p=0,258	p=0,249	p=0,048	p<0,001
Ninguna	104 (4,2)	51 (2,2)	249 (9,4)	229 (9,3)	930 (31,2)	895 (31,7)	869 (38,4)	730 (34,2)
	3,4-5,0	1,6-2,8	8,3-10,5	8,2-10,5	29,5-32,8	30,0-33,4	36,4-40,4	32,2-36,2
Leve-moderada	13 (6,0)	13 (2,9)	26 (9,1)	50 (8,4)	96 (34,5)	176 (35,1)	292 (42,8)	366 (42,6)
	2,8-9,1	1,3-4,4	5,8-12,4	6,2-10,6	28,9-40,1	30,9-39,2	39,0-46,5	39,3-45,9
Moderada-grave	4 (7,8)	7 (5,3)	3 (4,8)	7 (4,6)	15 (40,5)	33 (35,9)	55 (45,8)	110 (47,4)
	0,5-15,2	1,5-9,1	0-10,2	1,3-7,9	24,7-56,4	26,1-45,7	36,9-54,7	41,0-53,8
*Cobertura sanitaria*								
			p=0,096	p<0,001	p<0,001	p=0,050	p=0,037	p<0,001
Pública			217 (9,0)	216 (8,3)	832 (30,8)	873 (31,3)	965 (39,0)	920 (35,5)
			7,8-10,1	7,2-9,3	29,0-32,5	29,5-33,0	37,1-40,9	33,7-37,4
Privada			5 (5,8)	7 (5,5)	19 (20,4)	27 (35,1)	30 (33,3)	31 (34,8)
			0,9-10,8	1,5-9,4	12,2-28,6	24,4-45,7	23,6-43,1	24,9-44,7
Ambas			60 (12,0)	66 (13,9)	192 (38,3)	204 (37,1)	254 (45,0)	286 (46,7)
			9,1-14,8	10,8-17,0	34,1-42,6	33,1-41,1	40,9-49,1	42,8-50,7
Otros			0	0	1 (10,0)	1 (25,0)	8 (38,1)	3 (17,6)
			-	-	0-28,6	0-67,4	17,3-58,9	0-35,8
*Barreras atención médica*								
			p=0,544	p=0,432	p<0,001	p<0,001	p<0,001	p<0,001
Sí			36 (9,1)	54 (10,0)	205 (46,9)	233 (42,0)	345 (49,4)	412 (49,6)
			6,3-12,0	7,5-12,6	42,2-51,6	37,9-46,1	45,7-53,1	46,2-53,0
No			188 (10,1)	177 (8,9)	755 (30,0)	795 (30,8)	811 (37,6)	746 (33,8)
			8,8-11,5	7,7-10,2	28,2-31,8	29,1-32,6	35,5-39,6	31,9-35,8

IC95%: intervalo de confianza al 95%. Valores de p obtenidos mediante la prueba de Chi-cuadrado, excepto en caso de frecuencias esperadas pequeñas, que se aplicó la prueba exacta de Fisher. Los espacios en blanco sin datos indican que dicha información no estaba disponible para el año de encuesta.

En todos los años de encuesta, salvo 2014, los porcentajes más altos de realización de SOH en ambos sexos se encontraron entre quienes percibían su salud como negativa, padecían alguna enfermedad crónica, limitaciones diarias o problemas de salud mental (Tabla 5). En cuanto a la cobertura sanitaria, quienes se realizaron la prueba en mayor proporción fueron hombres y mujeres con cobertura sanitaria mixta (pública y privada). La percepción de barreras a la atención médica también comportó una mayor realización de la prueba, con diferencias significativas a partir de 2020.

## DISCUSIÓN

Este estudio muestra un cambio en la tendencia del porcentaje de realización de SOH tras los primeros años de implantación del cribado de CCR en España (2009-2014), período durante el cual el crecimiento anual fue más rápido, seguido de un aumento más lento entre 2014 y 2023. Asimismo, revela que los patrones desiguales de participación en las pruebas de cribado según el territorio (CCAA) y las características socioeconómicas, con menor participación en mujeres y en los grupos de edad más jóvenes, persisten a lo largo de todo el período de estudio.

Se ha puesto de manifiesto cómo los aumentos más llamativos en el porcentaje de realización de la prueba de detección precoz de CCR se ven en la encuesta posterior a la implantación del programa en cada comunidad. Además, la realización aumenta conforme transcurren más años de programa. Es por ello que, en 2020, cuando todas las CCAA han iniciado el programa, se observa un marcado aumento generalizado. Con todo, llama la atención que algunas de las comunidades con mayor recorrido de cribado mostraran un descenso en 2023 (Cantabria, Comunidad Valenciana, Navarra y País Vasco). Cabe resaltar el caso de la Comunidad Valenciana, que mantuvo el crecimiento hasta 2023, año en el que el porcentaje observado en mujeres descendió significativamente por debajo del valor esperado para dicha comunidad, mostrando un déficit en el análisis *post hoc* de residuos estandarizados. El impacto de la pandemia de COVID-19 explica en parte estas observaciones, ya que durante el periodo de confinamiento se suspendieron los programas de cribado en todas las CCAA^35^. Nuestros resultados concuerdan con estudios realizados en Europa y España, donde la pandemia supuso un declive en la participación en los cribados de cáncer, en particular entre las mujeres y las personas mayores^36^^,^^37^.

Se ha advertido una ralentización del incremento de la realización de la prueba a partir de 2014 en el análisis de tendencias temporales. Precisamente en 2014 entra en la cartera de servicios comunes del Sistema Nacional de Salud (SNS) el programa de cribado de CCR^38^ y todas las CCAA deben ofrecer el programa de cribado poblacional. Se fueron sumando nuevos territorios hasta ofrecerse en todas las comunidades desde 2020, con mayor o menor grado de cobertura de la población diana. Previamente, en 2003 el Consejo de la Unión Europea emitió la recomendación de desarrollar programas de cribado poblacional (de cérvix, mama y colorrectal)^39^; y, en 2009, la Estrategia en Cáncer del SNS introdujo el cribado de CCR con la indicación de realizar la prueba de SOH bienal en población de 50-69 años^25^. Así, entre 2009 y 2014 hubo un incremento muy elevado de la participación, ya que se partía de una situación de escasa participación y menor muestra, al estar implantado el programa en pocas CCAA.

La Estrategia en Cáncer del SNS fue actualizada en 2021, incluyendo entre sus objetivos y acciones impulsar medidas de sensibilización y mejorar la accesibilidad a los programas de cribado de CCR para aumentar la tasa de participación, fijando la meta a alcanzar en un mínimo del 65%^7^. Si bien han pasado más de 10 años desde que se iniciaron los primeros cribados, los resultados sugieren que todavía hay margen de crecimiento en la participación. Si se mantuviese constante el APC estimado, nos acercaríamos al objetivo de participación en 2028. La mayoría de países de la Unión Europea no han llegado al 65% de participación^10^. Nuestros resultados aumentan la evidencia publicada acerca de que, en países con diferencias regionales en la participación, el porcentaje global es bajo (como en Italia, con un 36%^40^ o España, con un 38,7% según nuestros resultados) en comparación con países en los que la participación es más uniforme en sus regiones (como Dinamarca, con un 67,1%^10^). Esto pone de manifiesto la pertinencia de analizar las desigualdades regionales en la realización de la prueba y tenerlas en cuenta en las estrategias nacionales.

Conviene recordar que la nueva Recomendación del Consejo de la Unión Europea sobre el cribado de cáncer amplió el intervalo de edad para la población diana hasta los 74 años^41^, por lo que las tasas de participación podrían verse modificadas.

En cuanto a las diferencias por sexo, partiendo de una participación significativamente mayor en hombres en 2009, se fueron equiparando los porcentajes en 2014 y 2020 hasta que, en 2023, vuelven a ser las mujeres quienes participan menos. Las crisis y catástrofes impactan más en la salud de grupos sociales vulnerabilizados, acrecentando las inequidades en salud y afectando en mayor medida a las mujeres^42^. Tanto 2009 como 2023 fueron escenario de las consecuencias de dos crisis: la económica de 2007-2008 y la pandemia de COVID-19 entre 2020 y 2023.

De acuerdo con la literatura existente, encontramos gradientes socioeconómicos que comportan una mayor realización de la prueba en personas con altos niveles de estudios y de ingresos^13^^,^^15^^,^^16^ y que se mantienen a lo largo de todo el periodo. Esto muestra cómo, a pesar de la existencia en España de cobertura sanitaria universal y las campañas de sensibilización realizadas para fomentar la participación en los cribados, persisten las inequidades por nivel socioeconómico en la realización de las pruebas de cribado de CCR. En este punto conviene señalar que existen grupos poblacionales no cubiertos por los programas y que varían según CCAA, ya que cada una obtiene los datos de la población diana de fuentes diferentes (tarjeta sanitaria, padrón, censo). Por ejemplo, la Comunidad Valenciana y el País Vasco tienen una de las coberturas más amplias, incluyendo población no empadronada, reclusa o inmigrante^43^.

Variables como la edad, la convivencia, un estilo de vida saludable (actividad física, alimentación saludable y sin tabaco), una peor salud autopercibida, enfermedades crónicas, limitaciones diarias o problemas de salud mental, se relacionan con mayor participación, en la línea de estudios previos^10^^,^^13^^-^^16^. Un mayor contacto con el sistema sanitario y una mayor percepción del riesgo de padecer cáncer entre quienes padecen ya alguna patología podrían explicar la mayor participación, como manifiestan algunos estudios en los que una mayor frecuencia de visitas médicas se relaciona con una mayor participación en cribados de cáncer^44^.

Es llamativa la mayor realización de SOH entre quienes detectan problemas en su entorno urbano. No obstante, en la literatura se ha visto que estos se relacionan con barreras para un estilo de vida saludable, especialmente en personas mayores^45^.

Este estudio presenta algunas limitaciones, además de las inherentes a los estudios transversales, que no permiten inferir causalidad. Las preguntas acerca de la realización de la prueba de SOH en las encuestas utilizadas no siempre recogen el motivo de realización, ni se especifica en el cuestionario que se refieran a pruebas dirigidas a población asintomática, además de ser autodeclaradas, dificultando conocer si realmente se han realizado como prevención secundaria. Por ello, utilizar dichas preguntas como *proxy* de la participación en el cribado de CCR puede suponer una limitación y una fuente de sesgos de memoria o de deseabilidad social, entre otros. Adicionalmente, el hecho de que las respuestas sobre realización de la SOH en las encuestas estudiadas permitan incorporar las pruebas realizadas de forma oportunista o privada, puede explicar el hallazgo de una mayor participación entre los hombres, a diferencia de la mayor participación en mujeres que apuntan los informes de programas de cribado españoles^43^. Por último, se ha considerado pertinente utilizar todas las Encuestas Europeas de Salud en España junto con la encuesta de 2023, y no las Encuestas Nacionales, con el fin de otorgar coherencia a la comparabilidad de variables entre diferentes años y otros países europeos. No obstante, la estimación de modelos de regresión *joinpoint* con cuatro marcas temporales comporta algunas limitaciones en la robustez e interpretación de resultados^46^ que, si bien ofrecen indicios relevantes, debe realizarse con mesura.

En conclusión, este estudio descriptivo supone un punto de partida para orientar futuras investigaciones sobre los factores que influyen en la participación en el cribado de CCR con mayor profundidad, al tiempo que actualiza la literatura con los datos de la ESdE 2023. Aunque no se encontraron patrones claramente diferenciados por sexo, los hombres se realizaron más la prueba. Esto hace patente la necesidad de profundizar en los análisis con perspectiva interseccional y de género para explicar y abordar la diferente participación en mujeres y hombres. En esta línea, no puede dejarse de lado la reflexión sobre la precisión y disponibilidad de las fuentes de información, puesto que las encuestas (autodeclaradas) y los registros de cribado (participación real) aportan información valiosa, pero complementaria. Futuras investigaciones que contemplen el cruce de ambos podrán proporcionar resultados de notable interés. Será fundamental incluir los motivos de no participación, así como conocer el perfil de las personas que no participan, para diseñar intervenciones dirigidas y lograr aumentar la participación en el programa de cribado de CCR.

Estrategias como unificar tanto las fuentes de obtención de la población diana como los criterios de inclusión entre todas las CCAA podrían contribuir a mejorar la participación y reducir las desigualdades. Además, sería conveniente promover campañas de difusión del programa de cribado dirigidas especialmente a personas sanas con menor contacto con el sistema sanitario. Y, por otro lado, aprovechar los contactos sanitarios por otros motivos (consultas de Atención Primaria, vacunaciones, realización de otros cribados o cuando acompañan a otras personas) para recomendar activamente el cribado de CCR en mujeres en edad de participación. Esta misma estrategia, aplicada en personas de 50-54 años, con bajo nivel educativo o socioeconómico, que viven solas, con bajo apoyo social o con hábitos poco saludables, favorecería la participación de los perfiles menos implicados.

## Data Availability

Las bases de datos a partir de las cuales se ha desarrollado este trabajo son de acceso público en la página web del Instituto Nacional de Estadística (https://www.ine.es/).

## References

[1] European Cancer Inequalities Registry (2025). Perfiles Nacionales de Cáncer 2025.

[2] Red Española de Registros de Cáncer (REDECAN) (2025). Estimaciones de la incidencia del cáncer en España.

[3] Sociedad Española de Oncología Médica (SEOM) Las cifras del cáncer en España 2024.

[4] Marzo-Castillejo M, Bartolomé-Moreno C, Bellas-Beceiro B, Melús-Palazón E, Vela-Vallespín C (2022). Recomendaciones de Prevención del Cáncer. Actualización PAPPS 2022. Aten Primaria.

[5] Organización Mundial de la Salud, Agencia Internacional de Investigación sobre el Cáncer Código Europeo contra el Cáncer. Doce formas de reducir el riesgo de cáncer.

[6] Sociedad Española de Oncología Médica (SEOM) Situación del cribado de cáncer de colon en España. Marzo 2024.

[7] Ministerio de Sanidad Estrategia en cáncer del Sistema Nacional de Salud. Actualización aprobada por el Consejo Interterritorial del Sistema Nacional de Salud, el 24 de febrero de 2021.

[8] World Health Organization (2010). A conceptual framework for action on the social determinants of health. Social Determinants of Health Discussion Paper 2.

[9] Pedrós Barnils N, Gustafsson PE (2025). Intersectional inequities in colorectal cancer screening attendance in Sweden: Using decision trees for intersectional matrix reduction. Soc Sci Med.

[10] Ola I, Cardoso R, Hoffmeister M, Brenner H (2024). Utilization of colorectal cancer screening tests across European countries: A cross-sectional analysis of the European Health Interview Survey 2018-2020. Lancet Reg Health Eur.

[11] Cardoso R, Guo F, Heisser T, Hoffmeister M, Brenner H (2020). Utilisation of colorectal cancer screening tests in European countries by type of screening offer: Results from the European Health Interview Survey. Cancers (Basel).

[12] Zeng-Zhang L, de Miguel-Diez J, López-de-Andrés A, Jiménez-García R, Ji Z, Meizoso-Pito O (2024). Adherence to screening tests for gynaecological and colorectal cancer in patients with diabetes in Spain: A population-based study (2014-2020). J Clin Med.

[13] Portero de la Cruz S, Cebrino J (2023). Uptake patterns and predictors of colorectal cancer screening among adults resident in Spain: A population-based study from 2017 to 2020. Front Public Health.

[14] Nouni-García R, Lara-López Á, Carratalá-Munuera C, Gil-Guille VF, López-Pineda A, Orozco-Beltrán D (2022). Factors associated with colorectal cancer screening in Spain: Results of the 2017 national health survey. Int J Environ Res Public Health.

[15] Zamorano-Leon JJ, López-de-Andres A, Álvarez-González A, Maestre-Miquel C, Astasio-Arbiza P, López-Farree A (2020). Trends and predictors for the uptake of colon cancer screening using the fecal occult blood test in Spain from 2011 to 2017. Int J Environ Res Public Health.

[16] Cobo-Cuenca AI, Laredo-Aguilera JA, Rodríguez-Borrego MA, Santacruz-Salas E, Carmona-Torres JM (2019). Temporal trends in fecal occult blood test: Associated factors (2009-2017). Int J Environ Res Public Health.

[17] Bacigalupe A, Cabezas A, Bueno MB, Martín U (2020). El género como determinante de la salud mental y su medicalización. Informe SESPAS 2020. Gac Sanit.

[18] Cabanillas-Montferrer T, Giménez-Bonafé P (2022). El sesgo de género en la asistencia sanitaria: definición, causas y consecuencias en los pacientes. MUSAS Revistas Investigación en Mujer Salud y Sociedad.

[19] Berner AM, Lund J, Saunders CL (2021). Tackling the complexity of gender bias in primary care. Br J Gen Pract.

[20] Instituto Nacional de Estadística Encuesta Europea de Salud en España 2020. Metodología.

[21] Instituto Nacional de Estadística Encuesta de Salud de España 2023. Metodología.

[22] Instituto Nacional de Estadística Encuesta Europea de Salud en España 2014. Metodología.

[23] Instituto Nacional de Estadística Encuesta Europea de Salud en España 2009. Metodología.

[24] Instituto Nacional de Estadística Encuesta Europea de Salud en España. Resultados.

[25] Ministerio de Sanidad y Política Social Estrategia en Cáncer del Sistema Nacional de Salud. Actualización aprobada por el Consejo Interterritorial del Sistema Nacional de Salud, el 22 de octubre de 2009.

[26] Davinson KW, Barry MJ, Mangione CM, Cabana M, Caughey AB, US Preventive Services Task Force (2021). Screening for colorectal cancer: US Preventive Services Task Force recommendation statement. JAMA.

[27] United Nations. Department of Economic and Social Affairs (2019). World Urbanization Prospects. The 2018 Revision.

[28] Diaz T, Strong KL, Cao B, Guthold R, Moran AC, Moller A (2021). A call for standardised age-disaggregated health data. Lancet Healthy Longev.

[29] Domingo-Salvany A, Bacigalupe A, Carrasco JM, Espelt A, Ferrando J, Borrell C (2013). Propuestas de clase social neoweberiana y neomarxista a partir de la Clasificación Nacional de Ocupaciones 2011. Gac Sanit.

[30] Kocalevent RD, Berg L, Beutel ME, Hinz A, Zenger M, Härter M (2018). Social support in the general population: standardization of the Oslo social support scale (OSSS-3).. BMC Psychol.

[31] IPAQ Research Committee Guidelines for data processing and analysis of the International Physical Activity Questionnaire (IPAQ). April 2004.

[32] Fernandez-Navarro P, Aragones MT, Ley V (2018). Leisure-time physical activity and prevalence of non-communicable pathologies and prescription medication in Spain. PLos One.

[33] Finger JD, Tafforeau J, Gisle L, Ziese T, Thelen J, Mensink GBM (2015). Development of the European Health Interview Survey - Physical Activity Questionnaire (EHIS-PAQ) to monitor physical activity in the European Union. Arch Public Health.

[34] National Cancer Institute, Division of Cancer Control & Population Sciences, Surveillance Research Program (2025). Joinpoint Trend Analysis Software.

[35] Ministerio de Sanidad. Estrategia en Cáncer del Sistema Nacional de Salud (2023). Estudio de impacto de la pandemia por COVID-19 sobre la prevención y el control del cáncer en el Sistema Nacional de Salud 2022.

[36] Muschol J, Strauss C, Gissel C (2023). COVID-19 related decline in cancer screenings most pronounced for elderly patients and women in Germany: a claims data analysis. J Cancer Res Clin Oncol.

[37] Garrido-Cantero G, Longo F, Hernández-González J, Pueyo A, Fernández-Aparicio T, Dorado JF (2023). Impact of the COVID-19 pandemic on cancer diagnosis in Madrid (Spain) based on the RTMAD tumor registry (2019-2021). Cancers (Basel).

[38] Ministerio de Sanidad, Servicios Sociales e Igualdad (2014). Orden SSI/2065/2014, de 31 de octubre, por la que se modifican los anexos I, II y III del Real Decreto 1030/2006, de 15 de septiembre, por el que se establece la cartera de servicios comunes del Sistema Nacional de Salud y el procedimiento para su actualización. Boletín Oficial del Estado.

[39] Unión Europea (2003). Recomendación del Consejo de 2 de diciembre de 2003 sobre el cribado del cáncer. Diario oficial de la Unión Europea.

[40] Organisation for Economic Co-operation and Development (OECD) EU Country Cancer Profile: Italy 2023.

[41] Unión Europea (2022). Recomendación del Consejo de 9 de diciembre de 2022 relativa a la mejora de la prevención mediante la detección precoz: Un nuevo enfoque de la UE para el cribado del cáncer en sustitución de la Recomendación 2003/878/CE. Diario Oficial de la Unión Europea.

[42] Krieger N (2024). Advancing gender transformative intersectional science for health justice: An ecosocial analysis. Soc Sci Med.

[43] Red de Programas de Cribado de Cáncer Programas de cribado de cáncer colorrectal. Informe de Evaluación 2021.

[44] López Salas M, De Haro-Gázquez D, Fernández Sánchez B, Amador Muñoz ML (2023). Knowledge, compliance, and inequities in colon cancer screening in Spain: An exploratory study. Healthcare (Basel).

[45] Wood GER, Pykett J, Daw P, Agyapong-Badu S, Banchoff A, King AC (2022). The role of urban environments in promoting active and healthy aging: A systematic scoping review of citizen science approaches. J Urban Health.

[46] Ingram DD, Malec DJ, Makuc DM, Kruszon-Moran D, Gindi RM, Albert M, National Center for Health Statistics (2018). Guidelines for Analysis of Trends. Data Evaluation and Methods Research. Vital and Health Statistics.

